# Strategic Modulation of Isoniazid Solubility through Cocrystal Formation for Long-Acting Microneedle Therapy of Tuberculosis

**DOI:** 10.1021/acsami.5c13207

**Published:** 2025-09-04

**Authors:** Octavio E. Fandiño, Lucia K. McPeake, Huanhuan Li, Yaocun Li, Ryan F. Donnelly

**Affiliations:** School of Pharmacy, 1596 Queen’s University Belfast, Medical Biology Centre, 97 Lisburn Road, Belfast BT9 7BL, U.K.

**Keywords:** dissolving microneedles, transdermal, cocrystal, long-acting, tuberculosis

## Abstract

Tuberculosis (TB), caused by *Mycobacterium tuberculosis*, remains a global health emergency, particularly in low- and middle-income countries. Despite effective pharmacotherapy, prolonged treatment, poor adherence, and drug resistance continue to hinder eradication. Isoniazid (ISZ), a first-line antitubercular drug, is effective but limited by high aqueous solubility and short half-life, necessitating daily administration and causing plasma fluctuations. Considering these limitations, strategies to modulate ISZ solubility without altering pharmacodynamics are therefore of therapeutic interest. In this study, we report the design, synthesis, and characterization of a cocrystal of ISZ with salicylic acid (SA), a GRAS-status coformer with low solubility. Cocrystallization was employed to reduce ISZ solubility, enhancing its potential for sustained release. The ISZ–SA cocrystal was confirmed as a distinct crystalline phase by FTIR, DSC, and PXRD, and subsequently incorporated into dissolving microneedle array patches (MAPs) fabricated from biocompatible polymers via aqueous casting. These MAPs dissolve after skin insertion, releasing their load into the dermal microenvironment. FTIR confirmed the cocrystal’s structural integrity within the polymeric matrix, with no dissociation observed during formulation. *In vitro* release studies showed that ISZ–SA exhibited a slower, more sustained release compared to pure ISZ. *Ex vivo* dermatokinetic studies revealed significantly greater deposition of ISZ in epidermis (89%, 171.1 μg) and dermis (90%, 468.3 μg) with the cocrystal versus pure drug (36%, 210.0 μg). Enhanced dermal retention suggests localization within skin layers, acting as a depot for gradual systemic absorption. In contrast, pure ISZ permeated faster but deposited less, underscoring the cocrystal’s sustained delivery advantage. This work is among the first demonstrations of pharmaceutical cocrystals integrated into dissolving MAPs for transdermal delivery. Cocrystal engineering combined with MAPs may overcome inherent limitations of hydrophilic drugs like ISZ, enabling long-acting formulations that reduce dosing frequency, improve adherence, and enhance TB treatment outcomes, with potential application to other high-solubility drugs.

## Graphical abstract

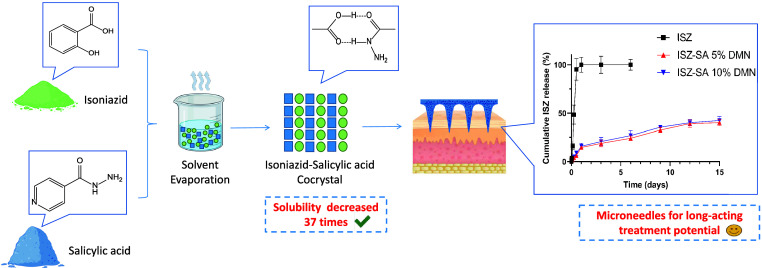

## 1 Introduction

Tuberculosis (TB), caused primarily by *Mycobacterium tuberculosis*, remains one of the top ten causes of death worldwide and the leading cause from a single infectious agent, surpassing even HIV/AIDS.^1,2^ Despite the availability of effective pharmacological treatments, TB continues to pose a substantial global health burden, particularly in low and middle income countries.^3^ Among the innumerable challenges faced in TB control efforts are prolonged treatment regimens, patient nonadherence, and the emergence of drug-resistant strains.^1^ The current end TB plan advocates for a comprehensive “health and beyond health” approach that aligns with the United Nations Sustainable Development Goals, recognizing that the underlying causes of tuberculosis extend beyond the health sector and necessitate coordinated multisectoral efforts. ISZ, a first-line antitubercular agent, is pivotal in both the prophylaxis and active treatment of TB.^2^ However, its therapeutic efficacy is closely tied to patient compliance, which is often undermined by the need for frequent oral dosing and systemic side effects. In this context, the development of long-acting drug delivery systems represents a transformative strategy for improving treatment outcomes and mitigating resistance.^4,5^ One of the critical of limitations of ISZ is its pharmacokinetic, because it has high aqueous solubility and short half-life, which necessitate daily oral administration.^6^ While this characteristic facilitates rapid absorption, it also leads to swift systemic clearance and fluctuating plasma concentrations, making sustained drug exposure difficult to maintain.^6^ The pharmacological profile of ISZ exhibits a formulation challenge; although it shows high bioavailability, its pronounced hydrophilicity limits the feasibility of developing long-acting or depot delivery systems. Accordingly, strategies that can modulate the solubility of ISZ without compromising its therapeutic activity are of considerable interest. Among these, cocrystallization has emerged as a promising and versatile approach.^7^ Pharmaceutical cocrystals are defined as multicomponent crystalline structures composed of an active pharmaceutical ingredient (API) and one or more coformers, typically held together via noncovalent interactions such as hydrogen bonding, π-π stacking, or van der Waals forces.^7–9^ Unlike salts or solvates, cocrystals retain the neutrality of the API and allow for fine-tuning of physicochemical properties without chemical modification of the molecule itself.^8,9^ Over the past decade, cocrystal engineering has been successfully employed to enhance solubility, stability, and bioavailability of poorly soluble drugs.^8^ However, the reverse process, modulating or even decreasing the solubility of highly soluble drugs, has gained less attention but is equally valuable, particularly in the context of long-acting drug formulations.^8^ In the case of ISZ, designing cocrystals with reduced aqueous solubility could serve to extend its release profile, thereby supporting the development of depot-like systems amenable to infrequent dosing. This work explores the design and characterization of novel ISZ cocrystal to reduce aqueous solubility, with the aim of enabling a long-acting and incorporate them to microarray patches for transdermal delivery system. Transdermal delivery, although traditionally limited by the barrier properties of the stratum corneum, has experienced a significant advancement with the emergence of microneedle (MN) technologies.^10–12^ Dissolving MN or microarray patches (MAP) offer a minimally invasive, painless, and patient-friendly platform for the delivery of drugs across the skin. These MAP are fabricated from biocompatible polymers that encapsulate the drug and dissolve upon skin insertion, releasing the drug load into the dermal microenvironment for systemic absorption.^13^ Incorporating poorly water-soluble cocrystals into dissolving MAP introduces a strategic advantage: the drug is retained in the epidermis or dermis in a controlled-release form, gradually dissolving over time, thus potentially achieving sustained therapeutic plasma levels from a single application. Such an approach could revolutionize the management of TB by reducing the frequency of administration, improving patient compliance, and diminishing the likelihood of resistance arising from missed doses. The objective of this work was the application of a combination of cocrystal engineering with transdermal MN delivery hinges on multiple interdisciplinary considerations to obtain a long-acting formulation with ISZ to the treatment of TB. The cocrystals must exhibit a robust reduction in aqueous solubility relative to ISZ, while maintaining chemical and thermal stability suitable for processing and storage. They must be compatible with the fabrication techniques of dissolving MAP, which typically involve aqueous or semiaqueous polymer solutions, and must not recrystallize or degrade during formulation. The mechanical integrity and insertion capability of the resulting MAP must be preserved, ensuring that the microneedles can truly penetrate the stratum corneum and deliver the cocrystals into the viable epidermis or dermis.

## 2 Material and Methods

### 2.1 Materials

ISZ also known as, Isonicotinic acid hydrazide, (purity, ≥98%), was for high provided by Alfa Aesar (Lancashire, UK). SA and Poly­(vinyl alcohol) (PVA) 9–10 kDa, were purchased from Sigma-Aldrich in Dorset, UK. On the other hand, poly­(vinylpyrrolidone) (PVP) 58 kDa, was supplied by Ashland in Kidderminster, UK. Sigma–Aldrich, Dorset, UK, provided to us reagents including sodium carbonate, phosphate-buffered saline (PBS) pH 7.4 tablets and methanol for high-performance liquid chromatography (HPLC). Elga Purelab DV25 water purification system (Veolia Water Systems, Ireland) was used to obtain HPLC-grade water. Acetone (ACE) and Ethanol Absolute (EtOH), both (≥99.8%, analytical reagent), were purchased from VWR Chemicals, Gdańsk, Poland.

### 2.2 Croystal Synthesis

#### 2.2.1 Coformer Selection

The selection of coformers is one of the most important steps in the cocrystal synthesis^14^ and was based on two main criteria. First, coformers were chosen for their ability to form hydrogen bonds with the functional groups present in ISZ, thereby favoring supramolecular interactions that could lead to cocrystal formation. Second, coformers with low aqueous solubility were prioritised, with the aim of reducing the overall aqueous solubility of ISZ when incorporated into a potential cocrystal.

#### 2.2.2 DSC as a Screening Method for Cocrystal Identification

To assess the potential for cocrystal formation between ISZ and the selected coformers (SA, para-aminobenzoic acid [PABA], and rifapentine [RIF]), binary physical mixtures (PM) were prepared in ISZ–coformer molar ratios of 1:1, 1:2, and 2:1. These mixtures were obtained by accurately weighing the components in the specified molar ratios into Eppendorf tubes, followed by vortex mixing for 15 s to ensure homogeneity. The resulting mixtures were analyzed by Differential Scanning Calorimetry (DSC), to evaluate which coformers and molar ratios showed evidence of potential cocrystal formation with ISZ, based on thermal behavior distinct from that of the individual components or simple PM.^15^

#### 2.2.3 Synthesis via Solvent Evaporation Crystallization

The synthesis of the cocrystals was carried out using solvent evaporation crystallization (CRYS) technique.

A. For the synthesis of cocrystals via CRYS, 500.0 mg (3.65 mmol) of ISZ and 503.6 mg (3.65 mmol) of SA were mixed with 80 mL of ethanol (EtOH) in a beaker, resulting in a suspension. To enhance the solubility of the compounds, the resulting suspension was subjected to sonication at 40 °C until complete dissolution was achieved, yielding a clear solution. The solution was then left to stand in a fume hood for 48 h until complete evaporation of the EtOH and the formation of a crystalline phase. The obtained samples were stored in light-protected glass vials within a vacuum desiccator containing silica gel as the desiccant

#### 2.2.4 Physical and Chemical Characterization by DSC, FTIR and PXRD

To confirm the formation of a crystalline phase between ISZ and the selected coformers, FTIR, DSC, and PXRD analyses were executed consequently. Differential scanning calorimetry (DSC) was used to analyze the thermal behavior of the samples obtained. The DSC analysis was performed using a DSC Q20 from TA Instruments (Elstree, Hertfordshire, UK). The experimental conditions were a temperature range of 25–300 °C, a heating rate of 10 °C/min, and a nitrogen flow rate of 50 mL/min. Spectroscopic characterization was conducted by means of Fourier transform infrared (FT-IR) spectroscopy. Measurements were obtained with a Spectrum Two FT-IR spectrometer (PerkinElmer, Waltham, MA), fitted with a MIRacle diamond attenuated total reflectance (ATR) accessory supplied by PIKE Technologies (Fitchburg, MA). Spectra were collected across the range of 4000–600 cm^–1^, using a resolution of 4 cm^–1^, and each spectrum represented the average of 32 scans. Powder X-ray diffraction (PXRD) was employed to determine if the solid forms obtained corresponded to new phases or not. Measurements were conducted at 20–25 °C utilizing a Bruker AXS D8 Advance diffractometer running at 40 kV and 40 mA with a Cu Kα radiation source (λ = 1.5418 Å). Data were collected over the 2θ range of 2–40° with a step size of 0.05°, while samples were continuously rotated at 30 rpm during acquisition. Prior to analysis, the powders were gently ground in an agate mortar and mounted on a silicon zero-background holder.

### 2.3 Preparation and Characterization of Dissolving MAPs

#### 2.3.1 Preparation of Dissolving MAPs

For the preparation of the dissolving microneedles, a blend of two polymeric stock solutions was employed. Stock solutions of PVP (58 kDa, 40% w/w) and PVA (9–10 kDa, 40% w/w) were prepared. The PVP stock solution was obtained by adding 20 g of polymer to 30 g of deionized water in a 50 mL Falcon tube, followed by manual stirring with a spatula until a homogeneous mixture was achieved. To ensure complete dissolution of the polymer, the resulting mixture was subjected to sonication for 24 h. In contrast, the PVA stock solution was prepared in a similar method; however, instead of sonication, the mixture was heated at 80 °C for 24 h to guarantee full polymer solubilization.The dissolving MAPs were produced using a double-casting method, in which the microneedle tips were loaded with a polymeric solution containing the cocrystal and pure ISZ, followed by the addition of a polymer-only baseplate (Figure 1). The final formulations prepared are detailed in Table 1. As shown in Table 1, two concentrations of PVA (9–10 kDa) and PVP (58 kDa) were employed, with different proportions of the cocrystal and pure ISZ used as controls for the release study. The microneedles were cast in circular silicone molds (pyramidal shape, 600 needles, 300 μm × 300 μm base width over an area of 0.75 cm^2^, with a height of 750 μm). Primarily, 100 mg of the polymer–cocrystal–drug mixture was dispensed into the molds to form the first layer. The molds were then placed in a pressure chamber at 4 bar for 15 min to ensure complete covering of the microneedle cavities. Excess material from the first layer was carefully removed with a spatula, and the molds were left to dry at room temperature for 24 h. Subsequently, 300 mg of the drug-free polymeric solution was added to form the second (baseplate) layer. To avoid air bubbles, the filled molds were centrifuged at 3500 rpm for 10 min. Finally, the formulations were left to dry at room temperature for 24 h, after which the patches were removed from the molds, and any excess edges were trimmed with scissors.

**Figure 1:**
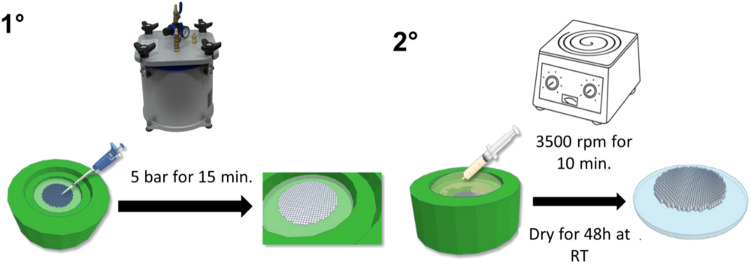
Schematic representation of the dissolving MAP manufacturing process.

**Table 1:** Dissolving MAP Formulation

dissolving MAP formulation	drug or cocrystal (% w/w)	PVA 9–10 kDa (% w/w)	PVP 58 kDa (% w/w)
ISZ	5	20	20
ISZ-SA 5%	5	20	20
ISZ-SA 10%	10	20	20

#### 2.3.2 Mechanical Strength and Insertion Testing of Dissolving Microneedle Patch

An important parameter to consider in the mechanical characterization of microneedles is the comparison of needle height before and after compression, as this provides an indication of their mechanical strength and their ability to be inserted into the skin. For this evaluation, a TA-TX2 Texture Analyzer (TA, Stable Microsystems, Heslmere, UK) was employed in compression mode.^16^ The measurement of microneedle height before and after compression was performed visually using a Leica EZ4 D digital light microscope (Leica Microsystems, Milton Keynes, UK). The compression process was performed by applying a force of 32 N for 30 s. The eq 1 was used for the calculation of the percentage needle height, where the height before compression is represented by *H*0 and the height after compression by *H*1.

1 $$\mathrm{high}\;\mathrm{reduction}\;\left(\%\right)=\frac{\left(H0-H1\right)}{H1}\times 100$$

Another critical mechanical parameter for microneedles is their insertion capability into the skin. For this study, a validated testing model previously established in our laboratory was employed.^16^ This model simulated skin by using eight layers of Parafilm M, each with an approximate thickness of 126 μm. The test was conducted by mounting the dissolving MAPs onto the TA-TX2 Texture Analyzer in compression mode. The equipment was set to operate at a constant speed of 1.19 mm/s, applying a force of 32 N for 30 s. Upon completion of the insertion process, the MAPs were removed from the Parafilm M layers, and the perforations created in each layer were subsequently quantified using a Leica EZ4 D digital light microscope.The proportion of holes per layer, relative to the number of needles in the array, was calculated using eq 2.

2 $$\mathrm{holes}\;\mathrm{in}\;\mathrm{parafilm}\;M=\frac{\left(\mathrm{number}\;\mathrm{of}\;\mathrm{holes}\;\mathrm{observed}\right)}{\mathrm{number}\;\mathrm{of}\;\mathrm{MN}\;\mathrm{in}\;a\;\mathrm{array}}\times 100$$

Needle length measurements were performed using ImageJ software (National Institutes of Health, Bethesda MD).^16^

#### 2.3.3 Dissolution Time of Dissolving Microneedles

The dissolution time of each formulation in phosphate-buffered saline (PBS, pH 7.4) was assessed in order to determine how long the MAPs would require to dissolve upon application. For this study, three replicates of each formulation were placed in 20 mL glass vials, to which a magnetic stirring bar and 10 mL of dissolution medium (PBS, pH 7.4) were added. The experiment was conducted at 37 °C under continuous stirring at 1200 rpm. Dissolution time was determined visually, and the measurement was recorded using a digital timer.

#### 2.3.4 Drug Content within Dissolving MAPs

Each MAP was completely dissolved in 10 mL of PBS (pH 7.4) at 37 °C. For this purpose, the same procedure described in Section 2.3.3 Dissolution time of dissolving microneedles was followed. The resulting solutions were filtered using 0.2 μm Minisart filters, and appropriate dilutions were prepared to fit within the calibration curve range established for the HPLC method employed. The resulting samples were subsequently analyzed by HPLC to determine the ISZ content in each microneedle.

#### 2.3.5 Drug Quantification by High Performance Liquid Chromatography (HPLC)

ISZ was quantified using reverse-phase high-performance liquid chromatography (RP-HPLC) (Agilent Technologies 1200 Infinity compact LC Series), comprising an Agilent degasser, binary pump, auto standard injector, and a UV detector. An Phenomenex SphereClone C-18 (2.7 μm pore size, 4.6 mm × 150 mm) was used for chromatographic separation. The mobile phase consisted of MeOH and water in a 70:30 (v/v) ratio. The temperature used was 30 °C with a flow rate of 0.5 mL/min and an injection volume of 40 μL and a runtime of 8 min. The wavelength used for UV detection was 264 nm, previously validated in our group.^17^

#### 2.3.6 FTIR Analysis of ISZ–SA Cocrystal Dissolving MAPs

FTIR analysis was employed to determine whether the cocrystal incorporated into the dissolving MAPs remained intact or dissociated into its original components upon formulation. To this end, the microneedles from three cocrystal-loaded dissolving MAPs were carefully removed using a scalpel. The isolated needles were then gently ground using a mortar and subsequently analyzed by FTIR spectroscopy. The resulting spectra were compared to those of the pure ISZ–SA cocrystal and a PM of the polymers used in the MAP formulation, to assess the structural integrity of the cocrystal within the polymeric matrix.

#### 2.3.7 *In Vitro* Release Study

A dialysis membrane method was used to assess the *in vitro* release study by comparing pure ISZ with the dissolving MAPs loaded with ISZ cocrystals. The media used was phosphate buffer with pH 7.4. Sink conditions in this study were maintained using the solubility of ISZ determined in the previous section. Dissolving MAP of pure ISZ (∼0.7 mg/patch) and dissolving MAPs ISZ-SA 5 and 10% were placed in dialysis membrane bags previously activated in the medium employed. The system was closed using plastic clips and placed at 37 °C with agitation at 40 rpm in the incubator. One mL of samples was withdrawn at chosen time points (30 min, 1 h, 3, 6 h, 8 h, 1 day, 3 days, 6 days, 9 days, 12 days and 15 days) and replaced with fresh media, then quantified by HPLC method previous discribed. The results were obtained from measurements performed in triplicate (n = 3).

#### 2.3.8 *Ex Vivo* Dermatokinetic Study-Franz Cell

The *ex vivo* dermatokinetic study was conducted using Franz diffusion cells using porcine skin obtained from stillborn piglets by a scalpel. The removed skin was stored at −20 °C until use. The skin was fixed to the donor compartment of the Franz cells using ethyl-2-cyanoacrylate/propyl methacrylate adhesive, with the epidermal side facing upward. Insertion of the MAPs was achieved by applying manual pressure for approximately 30 s. To prevent the MAPs from being displaced from the skin, a cylindrical stainless-steel weight of 12 g was placed in each donor compartment. Phosphate-buffered saline (PBS, pH 7.4) was employed as the release medium, with 12 mL added to the receptor compartment of the Franz cells and maintained at 37 °C throughout the study. Sampling was performed at 0.5, 1, 3, 6, 8, and 24 h. All samples were centrifuged at 14,800 rpm for 30 min, analyzed using HPLC-UV, and diluted when necessary. The skin was carefully removed from the donor compartment at each time point and the epidermis and dermis were manually separated. To facilitate this process, the skin was heated at 60 °C for 5 min, after which the layers were separated using tweezers. The resulting samples were processed differently depending on the tissue type. The epidermis was homogenized in 2 mL of methanol using a Vortex mixer (Fisons Scientific Equipment, Leicestershire, UK) for 3 min. The dermis was processed using a Tissue Lyser LT (Qiagen Ltd., Manchester, UK); the tissue was placed in an Eppendorf tube, 500 μL of deionized water was added, and the sample was homogenized at 50 Hz for 15 min. Subsequently, 1 mL of methanol was added and a second homogenization cycle was performed. All homogenized samples, from both epidermis and dermis, were centrifuged at 14,000 rpm for 15 min and filtered through 0.2 μm Minisart filters prior to HPLC analysis. Experiments were conducted in triplicate (*n* = 3).

#### 2.3.9 Statistical Analysis

For the statistical analysis in this study, differences between the experimental groups were assessed using an analysis of variance (ANOVA) with a significance level set at *p* < 0.05. All regression and statistical analyses were performed using GraphPad Prism version 10.3 (GraphPad Software, San Diego, CA).

## 3 Results and Discussion

### 3.1 Coformer Selection and Cocrystal Screening *Via* Differential Scanning Calorimetry (DSC)

One of the most critical steps in cocrystal synthesis is the selection of an appropriate coformer and the subsequent screening for potential cocrystal formation between the selected coformers and ISZ. The coformers selected for this screening were SA, PABA, and RIF. Selection was based on two criteria: their low aqueous solubility and their ability to form hydrogen bonds with ISZ.Binary PMs were prepared for each selected coformer at the molar ratios described in Section 2.2.2 (Materials and Methods) and subjected to thermal analysis by DSC. Figure 2 showed the thermograms obtained for each coformer across all tested molar ratios. The DSC curves demonstrated that all three coformers were potential candidates for cocrystal formation with ISZ, with the 1:1 molar ratio showing the most favorable results. This conclusion was supported by the observation that the PMs displayed endothermic peaks at temperatures lower than the melting points of the pure components (Figure 2A–C), indicating eutectic phase formation followed by a subsequent endothermic event, potentially corresponding to the transformation of the metastable eutectic phase into a new crystalline phase, possibly a cocrystal.^18^ That such transitions are often accompanied by exothermic events indicating crystallization, though these may not always be detected depending on the DSC conditions employed.^18^ Known that cocrystals of PABA have already been reported,^19^ and considering its relatively higher aqueous solubility, it was excluded from further investigation. Furthermore, due to reported incompatibilities between RIF and ISZ and the known instability of RIF,^20^ this coformer was also excluded. Consequently, SA was selected as the optimal coformer for cocrystallization with ISZ in a 1:1 molar ratio.

**Figure 2:**
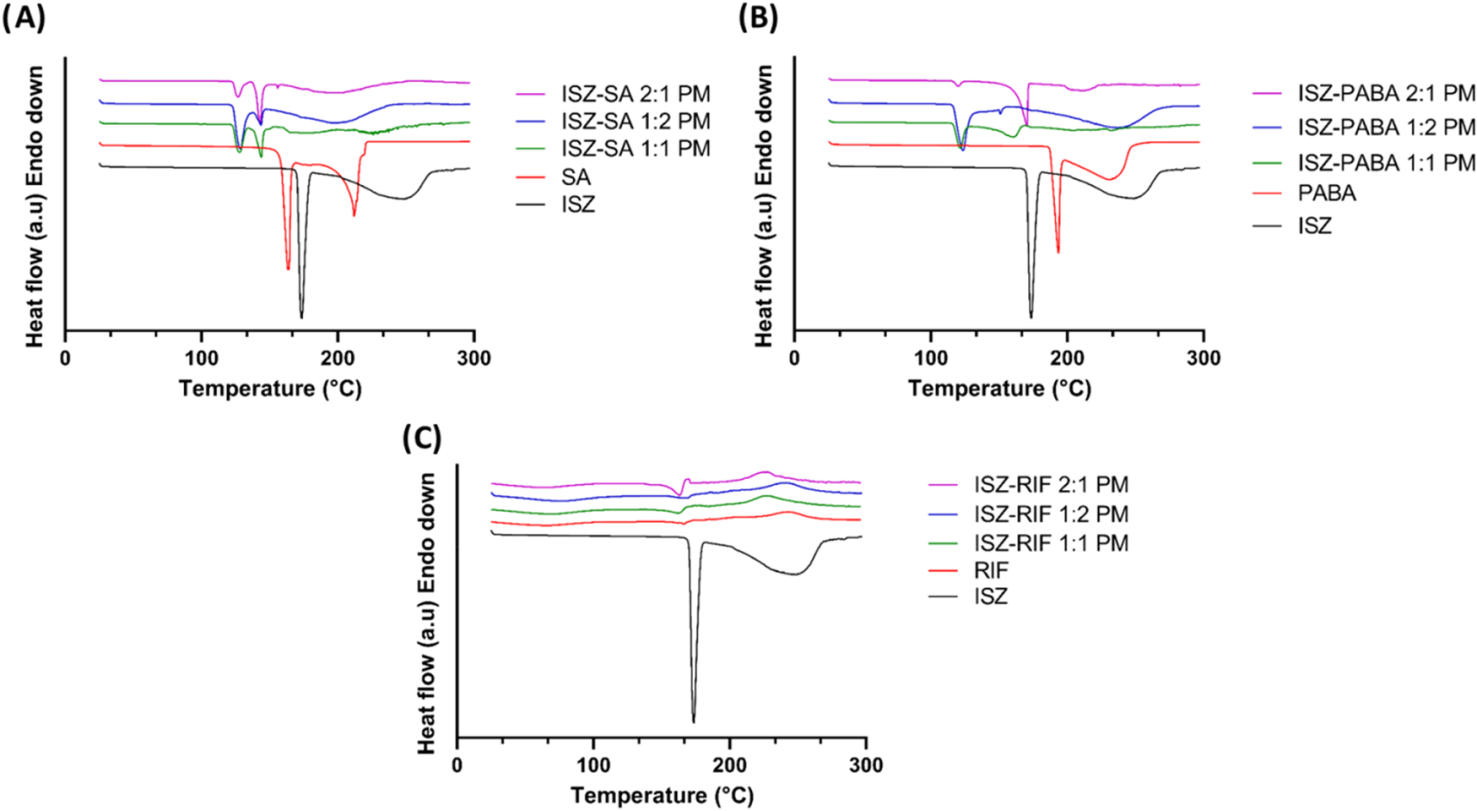
Representative DSC thermograms for ISZ, coformer selected (PABA, RIF and SA) and PMs of (A) ISZ; PABA; ISZ-PABA 1:1 PM; ISZ-PABA 1:2 PM and ISZ-PABA 2:1 PM (B) ISZ; RIF; ISZ-RIF 1:1 PM; ISZ-RIF 1:2 PM and ISZ-RIF 2:1 PM. (C) ISZ; SA; ISZ-SA 1:1 PM; ISZ-SA 1:2 PM and ISZ-SA 2:1 PM.

### 3.2 Synthesis of Cocrystal

Previous studies have reported supramolecular synthons involving the hydrazide group of ISZ and the carboxylic acid moiety of benzoic acid derivatives.^21^ Based on this, it was hypothesized that a synthon might form between the carboxylic group of SA and either the hydrazide group or the pyridinic nitrogen of ISZ (Figure 3A,B). Solvent evaporation methods were employed for cocrystal synthesis. This produced a crystalline product (ISZ-SA 1:1 CRYS) with acicular habit (Figure 3C). Crystals obtained were characterized using FTIR, DSC, and PXRD to assess molecular interactions and to confirm whether the obtained phases corresponded to cocrystalline structures.

**Figure 3:**
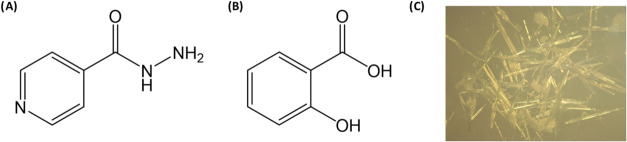
Representation of chemical structure of: (A) Representation of chemical structure of ISZ; (B) Representation of chemical structure of SA and (C) Microscopic images obtained using a Leica microscope showing the acicular crystals of the ISZ-SA 1:1 cocrystal synthesized in ethanol.

#### 3.2.1 Fourier-Transform Infrared Spectroscopy (FTIR)

FTIR spectra of the ISZ–SA cocrystals obtained *via* solvent evaporation is shown in Figure 4A, together with spectra of the pure starting materials. For ISZ, key absorption bands were observed in the 3500–2500 cm^–1^ region: a symmetric N–H stretch at 3303 cm^–1^ and an asymmetric N–H stretch at 3103 cm^–1^, corresponding to the NH_2_ group (Figure 4B). In the 2000–600 cm^–1^ region (Figure 4C), peaks at 1664 cm^–1^ and 1633 cm^–1^ were attributed to C=O stretching (amide carbonyl) and N–H bending, respectively.^22^ SA exhibited a broad, weak band at 3233 cm^–1^ corresponding to O–H stretching of the carboxylic acid group, overlapping with phenolic O–H stretching (Figure 4B). Additional significant peaks included symmetric C=O stretching of COO^–^ at 1651–1659 cm^–1^ and 1383 cm^–1^, C=C (phenolic) stretching at 1579–1610 cm^–1^, phenolic O–H bending at 1324 cm^–1^, and C–O (COO^–^) and C–OH stretching at 1294 cm^–1^ and 1154–1246 cm^–1^, respectively (Figure 4C), consistent with literature data.^23^ The FTIR spectrum of the ISZ–SA cocrystal revealed significant shifts and alterations in these characteristic bands (Figure 4A–C), suggesting interaction between the hydrazide group of ISZ and the hydroxyl and/or carboxylic groups of SA, supporting the formation of the hypothesized synthon shown in Figure 5. Detailed shifts are presented in Table 2.

**Figure 4:**
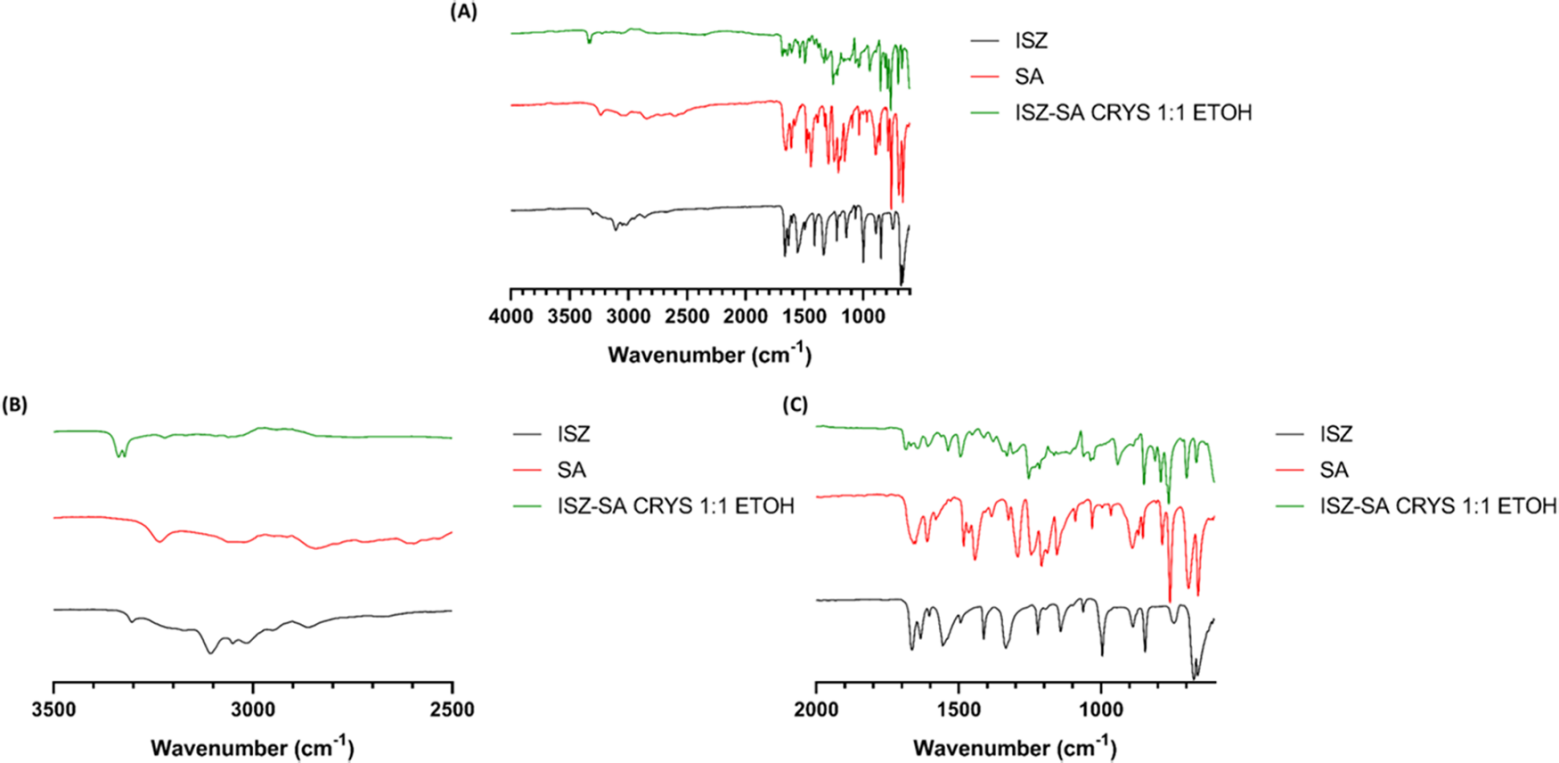
FTIR spectra for: (A) ISZ, SA and ISZ-SA 1:1 CRYS; (B) Magnified view of (A) in the region between 3500 and 2500 cm^–1^ and (C) Magnified view of (A) in the region between 2000 and 600 cm^–1^.

**Figure 5:**
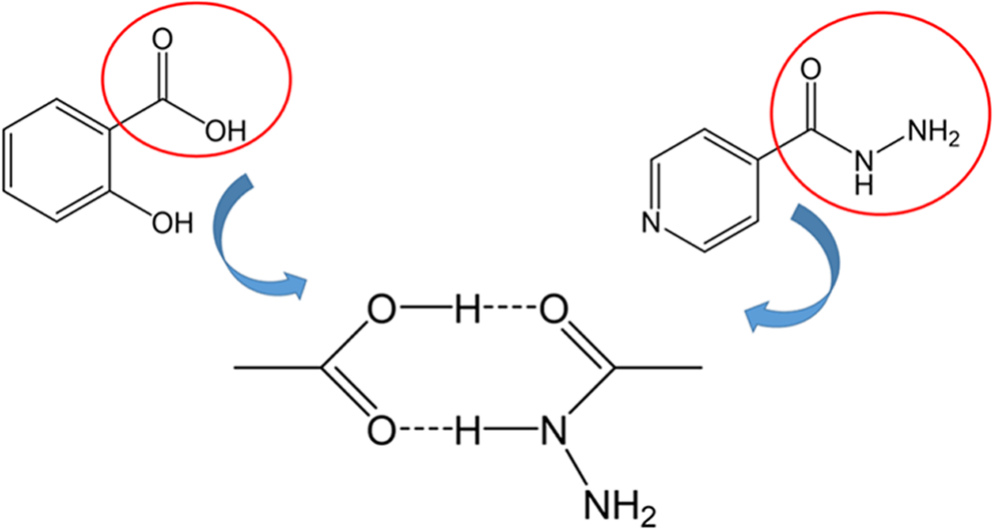
Schematic illustration of the proposed synthon between ISZ and SA.

**Table 2:** FTIR Absorption Values and Corresponding Functional Group Assignments

functional group	ISZ	SA	ISZ-SA1:1CRYS	comments
symmetric N–H stretching	3303		3338–3323	band shift and splitting in ISZ–SA 1:1 cocrystal
asymmetric N–H stretching	3103		3094	band shift in ISZ–SA 1:1 cocrystal
C=O stretching (amide)	1633		1641	band shift and decreased intensity in ISZ–SA 1:1 cocrystal
carboxylic O–H stretching		3233	3222	band shift in ISZ–SA 1:1 cocrystal
symmetric C=O stretching (carboxylic acid)		1659	1686	band shift and decreased intensity in ISZ–SA 1:1 cocrystal
		1651	1670	
		1383	1607	
Phenolic O–H bending		1324	1308	band shift and merging in ISZ–SA 1:1 cocrystal
		1294		

#### 3.2.2 Powder X-ray Diffraction (PXRD)

PXRD is the highest method to confirm the formation of new crystalline phases.^24^ The diffraction patterns of pure ISZ and SA matched those in the Cambridge Structural Database.^19,22^ Figure 6A shows the PXRD of ISZ-SA cocrystal in contrast with the correspondent PM and their pure components. The ISZ–SA cocrystal exhibited new diffraction peaks at 2θ = 8.28, 11.65, 16.64, 19.15, 20.38, 23.18, 24.72, and 27.96°, alongside the disappearance and merging of specific peaks present in the PM (Figure 6A,C). In contrast, 1:1 PM displayed the combined diffraction peaks of both components without any new reflections or peak shifts, indicating no spontaneous formation of a new solid phase (Figure 6B). These observations confirm the formation of a distinct crystalline phase, different from either component or a eutectic mixture, indicating a true cocrystal with both molecules present in the same crystal lattice.^25,26^

**Figure 6:**
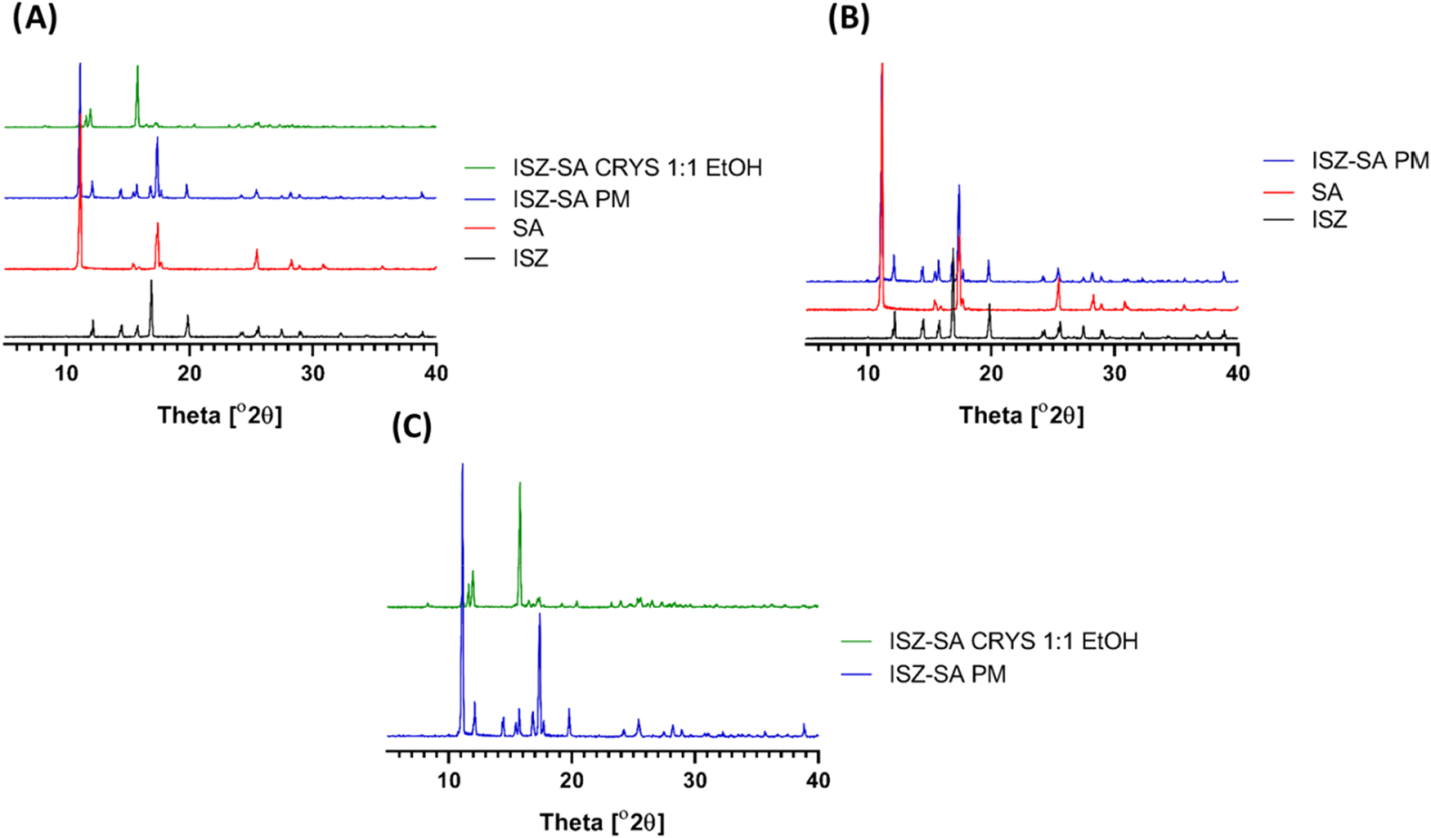
PXRD patterns of: (**A)** ISZ, SA, ISZ-SA 1:1 PM and ISZ-SA 1:1 CRYS; **(B)** ISZ, SA and ISZ-SA 1:1 PM and **(C)** ISZ-SA 1:1 PM and ISZ-SA 1:1 CRYS.

#### 3.2.3 Differential Scanning Calorimetry (DSC)

DSC analysis revealed that pure ISZ and SA melted at 173.4 and 163.6 °C, respectively, Figure 7i and ii, respectively. The ISZ–SA 1:1 cocrystal exhibited a single, sharp melting endotherm at 147.2 °C (Figure 7iv), lower than either of the pure components. While many cocrystals exhibit melting points between those of their components, it is not uncommon for them to have higher or lower melting points, depending on the nature of intermolecular interactions.^15^ Together with FTIR and PXRD data, described in Section 3.2.1 and 3.2.2 respectively, the presence of a unique melting point further supports the formation of a new crystalline phase. The presence of a single, sharp endothermic peak supports the hypothesis that a homogeneous, single-phase cocrystal was formed.

**Figure 7:**
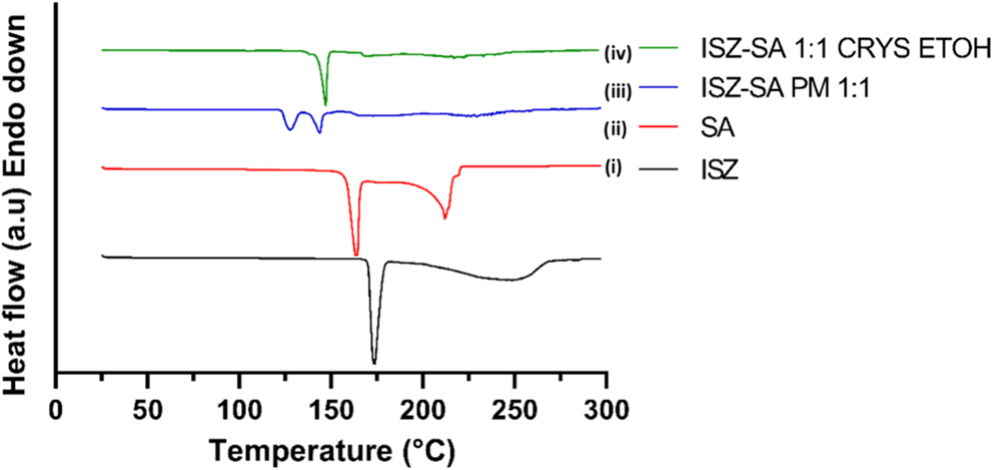
Representative DSC thermograms for: (i) ISZ; (ii) SA; (iii) ISZ-SA 1:1 PM and (iv) ISZ-SA 1:1 CRYS.

#### 3.2.4 Equilibrium Solubility Determination

The aqueous solubility of ISZ–SA 1:1 cocrystal synthesized via solvent evaporation was determined at 25 °C and compared to pure ISZ. The aqueous solubility of SA, taken from the literature, is 2.24 mg/mL.^27^ SA was selected specifically for its low solubility, aiming to reduce the high aqueous solubility of ISZ and develop a long-acting formulation. According to Good and Rodríguez-Hornedo,^28^ a solubility difference of approximately 10 times between drug and coformer is necessary to alter solubility via cocrystal formation. Experimental solubility values for ISZ were 138.80 ± 2.10 mg/mL in water and 146.4 ± 1.71 mg/mL in PBS pH 7.4, about 62 times higher than that of SA, thus fulfilling this requirement. The ISZ–SA cocrystal exhibited significantly reduced solubility: 3.71 ± 0.34 mg/mL in water and 4.67 ± 0.29 mg/mL in PBS. These values represent a 31- to 37 times reduction in solubility compared to pure ISZ, confirming that cocrystallization successfully modulated ISZ solubility.

### 3.3 Cocrystal-Loaded Dissolving MAPs: Preparation and Characterization

Dissolving microneedle arrays (MAPs) containing the ISZ-SA cocrystal were examined under a Leica EZ4D light stereomicroscope (Leica Microsystems, Milton Keynes, UK) after drying for 24 h at room temperature. Representative digital images are presented in Figure 8A. The microneedles exhibited a uniform pyramidal geometry with a height of approximately 750 μm. The consistent dimensions and regular interspacing of 50 μm are anticipated to ensure reproducible skin penetration while minimizing mechanical interference between adjacent needles. The sharp tips and pyramidal design confer mechanical robustness, enabling efficient insertion with minimal tissue damage. A previously validated microneedle insertion test was then employed to confirm the ability of the MAPs to effectively penetrate the stratum corneum.^18–21^ For this purpose, MAPs were applied to a stack of eight layers of Parafilm M, and a force of 32 N, representative of average human thumb pressure,^30^ was applied using a TA-TX2 Texture Analyzer. Figure 8B displays the insertion data, indicating that the MAPs successfully penetrated four layers of Parafilm M. Given that each layer has an approximate thickness of 126 μm, this corresponds to a minimum insertion depth of 630 μm, representing approximately 84% of the microneedle height. In addition to insertion studies, the mechanical strength of the MAPs was assessed. MAPs containing 5% ISZ-SA cocrystal (1:1 molar ratio) exhibited a statistically significant difference in microneedle height before and after the application of 32 N force (*p* = 0.0015, *p* < 0.05), although the reduction was less than 10% (specifically 3.33%), suggesting good mechanical properties (Figure 8C). Conversely, MAPs containing 10% cocrystal did not show a statistically significant height reduction postcompression (*p* > 0.05, Figure 8D), further supporting the conclusion that both formulations possessed adequate mechanical strength for effective skin insertion.

**Figure 8:**
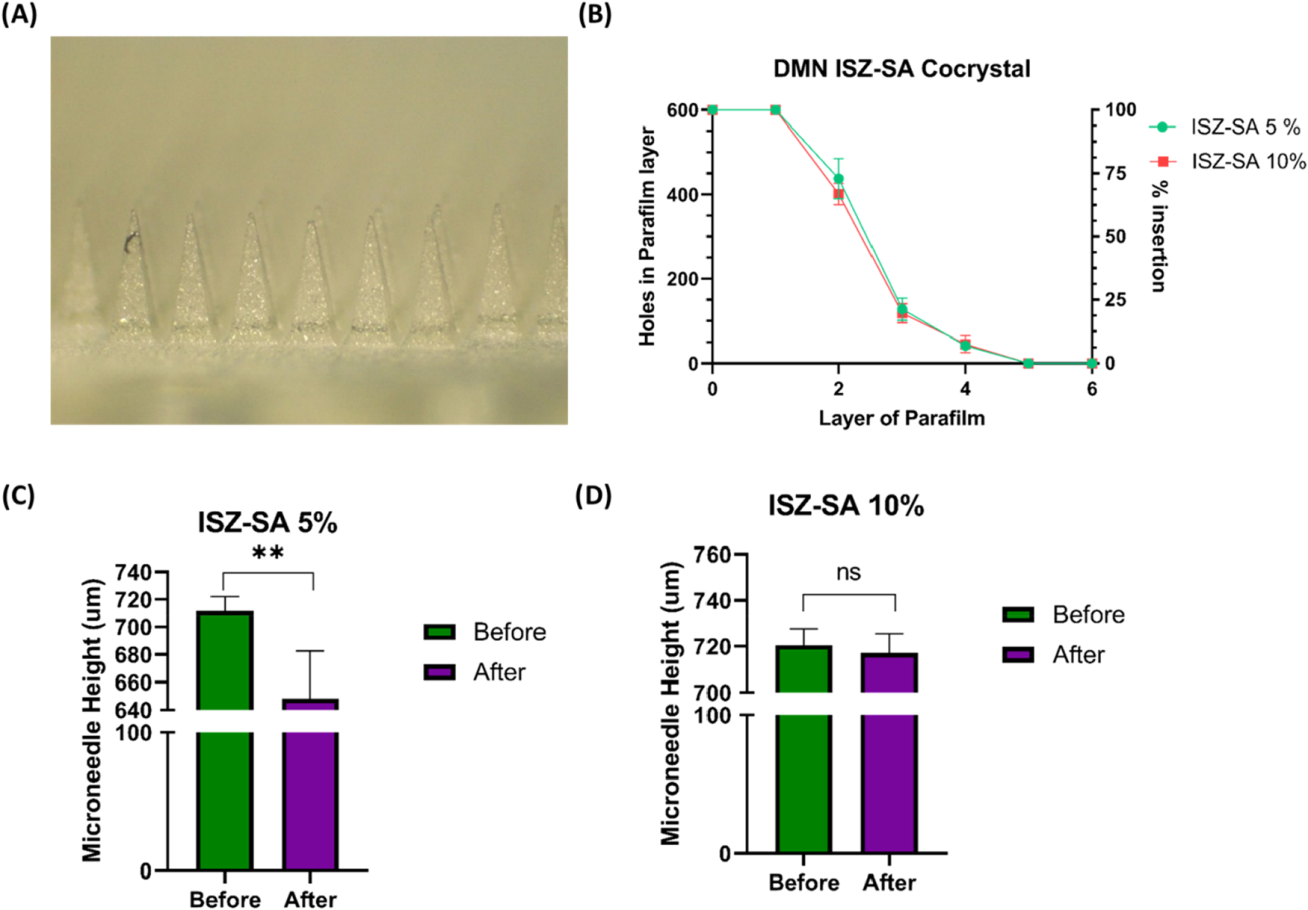
(A) Images of ISZ-SA 10% dissolving MAP; (B) Insertion depth for dissolving MAPs in Parafilm M layers and percentage of holes created in each layer (Means ± SD, *n* = 3); (C) Height reduction of dissolving MAPs before and after their insertion (32 N force for 30 s) into eight layers of Parafilm M, *p* value 0.0015, *p* value <0.05 and (D) Height reduction of dissolving MAPs before and after their insertion (32 N force for 30 s) into eight layers of Parafilm M, *p* value <0.05. (Means + SD, *n* = 6).

### 3.4 *In Vitr*o and *Ex Vivo* Characterization

#### 3.4.1 Dissolution Time and Drug Content

Dissolution times for the drug-loaded dissolving MAPs were determined by immersing each formulation in 10 mL of PBS (pH 7.4) at 37 °C and measuring the time required for complete dissolution. Table 3 summarizes the dissolution times and drug recovery results for all MAP formulations tested. No statistically significant differences were observed in drug recovery among the various MAPs (*p* > 0.05). However, the blank (nondrug-loaded) MAP dissolved approximately 270 s faster than the cocrystal-loaded MAPs, which was statistically significant (*p* < 0.05). The MAP loaded with pure ISZ dissolved only 70 s faster than the cocrystal-loaded MAPs, also a statistically significant difference (*p* < 0.05). This may be attributed to the reduced aqueous solubility of the cocrystal relative to pure ISZ.

**Table 3:** Drug Content and Dissolution Time for Blank, ISZ and ISZ-SA 1:1 CRYS^a^

dissolving MAPs	drug content in each patch (mg)	dissolution time formulation (s)
blank		515 ± 87
ISZ	0.68 ± 0.16	585 ± 71
ISZ-SA 1:1 CRYS 5%	0.28 ± 0.07	789 ± 85
ISZ-SA 1:1 CRYS 10%	0.56 ± 0.11	811 ± 94

a Means ± SD *n* = 3.

#### 3.4.2 FTIR Analysis of Cocrystal-Loaded Dissolving MAPs

To assess whether the cocrystal remained intact or dissociated upon incorporation into the MAP matrix, FTIR analysis was performed. Figure 9 displays the FTIR spectra of: (i) the pure ISZ-SA 1:1 cocrystal synthesized in ethanol, (ii) the 10% cocrystal-loaded MAP formulation, and (iii) a PM of the polymers used (PVA and PVP). The spectrum of the cocrystal-loaded MAPs retained the key characteristic absorption bands of the ISZ-SA cocrystal, indicating that the crystalline structure was largely preserved during formulation (Figure 9ii). However, some band broadening and overlapping were observed in the carbonyl (C=O) region. This is likely due to the strong interference from polymer-associated bands in the same region (Figure 9ii).^29,30^ On the other hand, the spectrum of the PM (Figure 9iii) showed no modification in the characteristic IR abortion bands, confirming that it is a simple physical blend with no chemical interactions between the components. Despite this, the data supports the conclusion that the ISZ-SA cocrystal was successfully incorporated into the MAP matrix without dissociation into its individual components.

**Figure 9:**
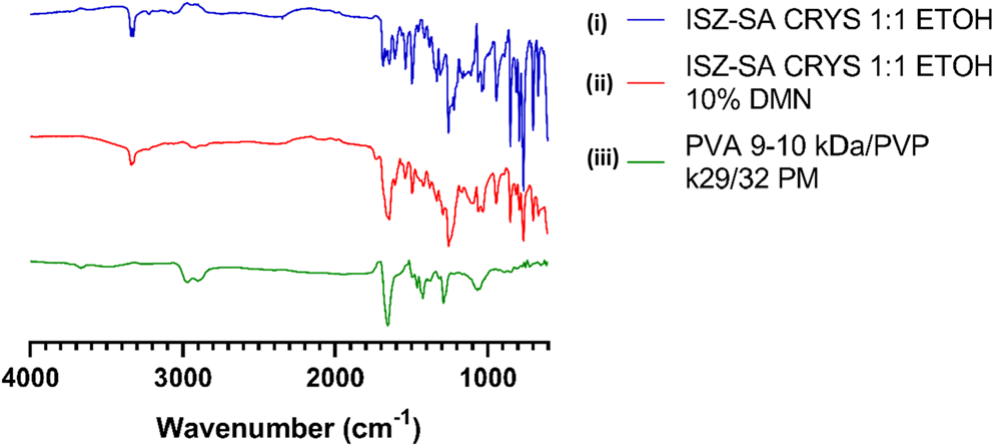
FTIR spectra for: (i) ISZ-SA 1:1 CRYS ETOH. (ii) ISZ-SA 1:1 CRYS ETOH 10% DMN. and (iii) PVA 9–10 kDa/PVP k29/32 PM.

#### 3.4.3 *In Vitro* Release Study

*In vitro* release study was conducted using a dialysis membrane method over 15 days. The release medium was phosphate buffer (pH 7.4), and drug concentrations were quantified by HPLC, as described in the Methods section. Figure 10 presents the release profiles of dissolving MAPs containing either pure ISZ or the ISZ-SA cocrystal. Both formulations exhibited similar, statistically indistinguishable release profiles (Figure 10ii,iii). The release was sustained and linear over the 15-day period, with no plateau reached, and more than 35% of the drug released by day 15 (Figure 10ii,iii). This suggests a continued release capability and a long-acting profile. In contrast, the pure ISZ-loaded dissolving MAPs released approximately 98% of the drug within the first 24 h, reaching complete release by day 3 (Figure 10i). Therefore, further monitoring beyond day 6 was deemed unnecessary. These findings indicate that cocrystallization effectively slowed the release of ISZ, achieving a sustained release profile. To assess the dissolution kinetics of the ISZ-SA cocrystal studied from dissolving MAPs, the *in vitro* release profiles obtained were fitted using different kinetic models. For this purpose, the models of Zero Order, First Order, Higuchi, and Korsmeyer-Peppas were employed. The ISZ 5% dissolving MAPs formulation exhibited a rapid release profile that followed First Order release kinetics (*R* ^2^ = 0.93), indicating that the release rate was concentration-dependent. However, the other formulations (ISZ-SA 5% and ISZ-SA 10% dissolving MAPs) were best described by the Higuchi model. They showed the highest regression coefficient values (greater than 0.98) in both cases, *R* ^2^ = 0.98 for ISZ-SA 5% dissolving MAP and *R* ^2^ = 0.99 for ISZ-SA 10% dissolving MAP, indicating that drug release was primarily governed by diffusion through the matrix.

**Figure 10:**
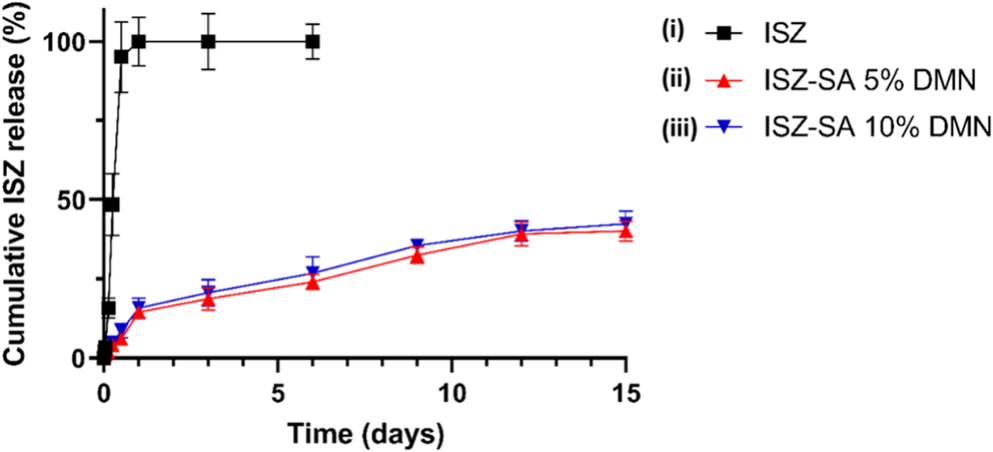
Cumulative release of: (i) ISZ; (ii) ISZ-SA 5% DMN and (iii) ISZ-SA 10% DMN.

#### 3.4.4 *Ex Vivo* Dermatokinetic Study

*Ex vivo* dermatokinetic study was performed to evaluate the skin penetration and retention of ISZ and its cocrystal using full-thickness neonatal porcine skin (∼500 μm) in modified Franz diffusion cells (Permegear, Hellertown PA). Figure 11 presents data on the skin deposition and permeation efficiency of ISZ and ISZ-SA cocrystal delivered *via* dissolving MAPs. Drug delivery efficiency (DE%) was calculated based on the initial ISZ loading in each MAP formulation. At the end of the 24-h study, the following DE% values were observed: ISZ 80% (544.2 μg), ISZ-SA cocrystal (5%) 72% (192.2 μg), and ISZ-SA cocrystal (10%) 76% (524.9 μg) (Figure 11A–C). For both cocrystal-loaded formulations, less than 5% of the drug was detected in the receptor compartment (Figure 11E,F), indicating that over 95% was retained within the skin layers, predominantly in the epidermis. This localized deposition may be due to the base of the microneedles remaining in direct contact with the epidermis during application. In contrast, pure ISZ exhibited increasing permeation over time, reaching approximately 40% in the receptor solution (Figure 11D). This behavior likely results from the higher aqueous solubility of pure ISZ, facilitating its faster diffusion through the skin compared to the more slowly dissolving cocrystal.

**Figure 11:**
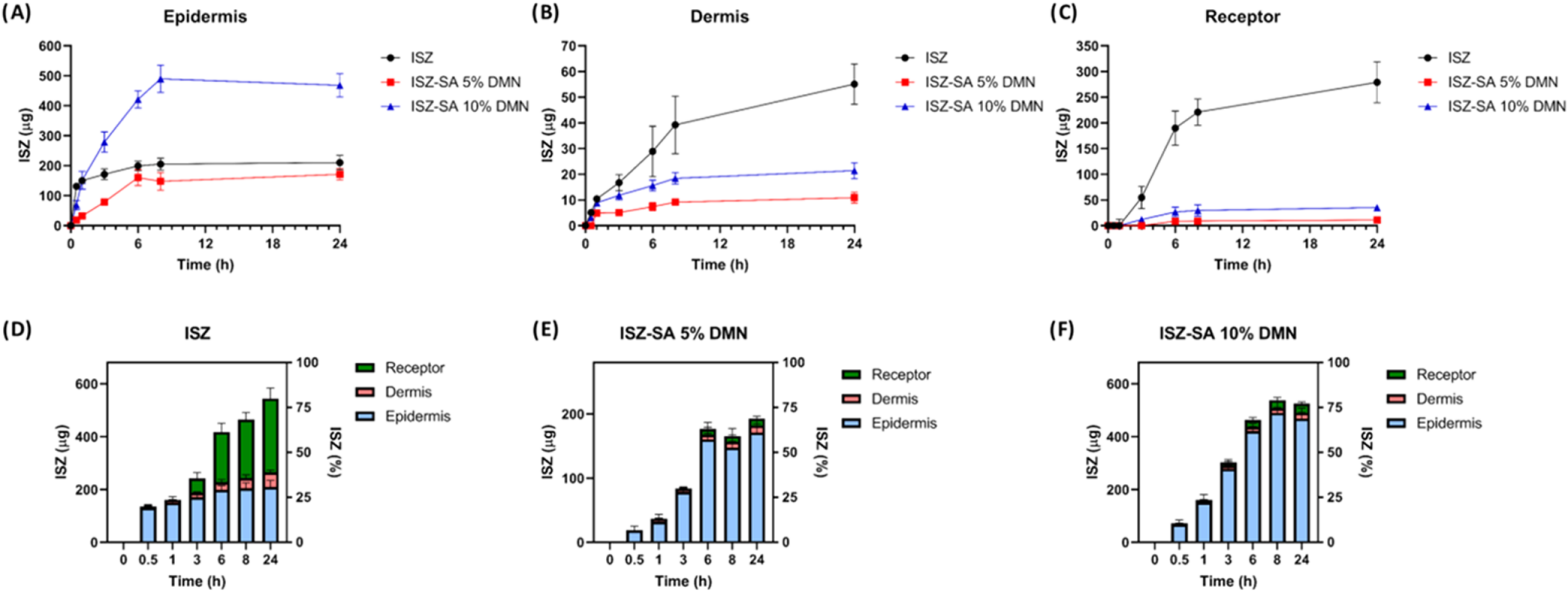
*Ex vivo* dermatokinetic study of dissolving MAPs across full-thickness neonatal porcine skin. (A, B): deposition profile of ISZ from all the formulations, in epidermis and dermis, respectively (means ± SD, *n* = 3). (C): skin permeation profile of ISZ from from all the formulations (means ± SD, *n* = 3). (D–F): sum of ISZ deposited in the skin (epidermis and dermis) and permeated through the skin from from all the formulations (means+SD, *n* = 3).

## 4 Conclusion

This study demonstrates that cocrystallization of ISZ with SA, followed by its incorporation into dissolving microneedle arrays, enables the development of a long-acting transdermal delivery system for tuberculosis treatment. The sustained release of ISZ from the cocrystal-loaded dissolving MAPs could potentially reduce dosing frequency, thereby improving patient compliance. *In vitro* and *ex vivo* studies clearly distinguished the release and permeation behaviors of the cocrystal and the pure drug, with the cocrystal showing enhanced retention in the skin layers and prolonged release. Notably, this work represents one of the first examples of utilizing pharmaceutical cocrystals in microneedle-based drug delivery systems. These findings highlight the promise of this novel formulation approach for the transdermal delivery of ISZ and potentially other poorly soluble therapeutic agents. Future work will focus on in vivo pharmacokinetic and therapeutic efficacy studies to confirm the long-acting behavior and clinical potential of this system.
